# Critically ill healthcare workers with the middle east respiratory syndrome (MERS): A multicenter study

**DOI:** 10.1371/journal.pone.0206831

**Published:** 2018-11-15

**Authors:** Sarah Shalhoub, Fahad Al-Hameed, Yasser Mandourah, Hanan H. Balkhy, Awad Al-Omari, Ghaleb. A. Al Mekhlafi, Ayman Kharaba, Basem Alraddadi, Abdullah Almotairi, Kasim Al Khatib, Ahmed Abdulmomen, Ismael Qushmaq, Ahmed Mady, Othman Solaiman, Abdulsalam M. Al-Aithan, Rajaa Al-Raddadi, Ahmed Ragab, Abdulrahman Al Harthy, Eman Al Qasim, Jesna Jose, Ghassan Al-Ghamdi, Laura Merson, Robert Fowler, Frederick G. Hayden, Yaseen M. Arabi

**Affiliations:** 1 Department of Medicine, Division of Infectious Diseases, University of Western Ontario, London, Canada, Department of Medicine, Division of Infectious Diseases, King Fahad Armed Forces Hospital, Jeddah, Saudi Arabia; 2 Department of Intensive Care, King Saud bin Abdulaziz University for Health Sciences, King Abdullah International Medical Research Center, King Abdulaziz Medical City, Jeddah, Saudi Arabia; 3 Department of Intensive Care Services, Prince Sultan Military Medical City, Riyadh, Saudi Arabia; 4 Department of Infection Prevention and Control, King Saud bin Abdulaziz University for Health Sciences, King Abdullah International Medical Research Center, King Abdulaziz Medical City, Riyadh, Saudi Arabia; 5 Department of Intensive Care, Alfaisal University, Dr Sulaiman Al-Habib Group Hospitals, Riyadh, Saudi Arabia; 6 Department of Critical Care, King Fahad Hospital, Ohoud Hospital, Al-Madinah Al-Monawarah, Saudi Arabia; 7 Department of Medicine, Alfaisal University, King Faisal Specialist Hospital and Research Center, Jeddah, Saudi Arabia; 8 Department of Critical Care Medicine, King Fahad Medical City, Riyadh, Saudi Arabia; 9 Intensive Care Department, Al-Noor Specialist Hospital, Makkah, Saudi Arabia; 10 Department of Critical Care Medicine, King Saud University, Riyadh, Saudi Arabia; 11 Intensive Care Department, King Saud Medical City, Riyadh, Saudi Arabia; 12 Intensive Care Department, King Faisal Specialist Hospital and Research Center, Riyadh, Saudi Arabia; 13 Intensive Care Department, King Abdulaziz Hospital, Al Ahsa, Saudi Arabia; 14 Department of Family and Community Medicine, King Abdulaziz University Hospital, Ministry of Health, Jeddah, Saudi Arabia; 15 Intensive Care Department, King Fahd Hospital, Jeddah, Saudi Arabia; 16 Department of intensive care, King Saud bin Abdulaziz University for Health Sciences, King Abdullah International Medical Research Center, King Abdulaziz Medical City, Riyadh, Saudi Arabia; 17 Infectious Diseases Data Observatory, Oxford University, Headiington, United Kingdom; 18 Department of Critical Care Medicine and Department of Medicine, Sunnybrook Hospital, Institute of Health Policy Management and Evaluation, University of Toronto, Toronto, Canada; 19 Department of Medicine, Division of Infectious Diseases and International Health University of Virginia School of Medicine, Charlottesville, Virginia, United States of America; The University of Hong Kong, CHINA

## Abstract

**Background:**

Middle East Respiratory Syndrome Coronavirus (MERS-CoV) leads to healthcare-associated transmission to patients and healthcare workers with potentially fatal outcomes.

**Aim:**

We aimed to describe the clinical course and functional outcomes of critically ill healthcare workers (HCWs) with MERS.

**Methods:**

Data on HCWs was extracted from a multi-center retrospective cohort study on 330 critically ill patients with MERS admitted between (9/2012–9/2015). Baseline demographics, interventions and outcomes were recorded and compared between survivors and non-survivors. Survivors were approached with questionnaires to elucidate their functional outcomes using Karnofsky Performance Status Scale.

**Findings:**

Thirty-Two HCWs met the inclusion criteria. Comorbidities were recorded in 34% (11/32) HCW. Death resulted in 8/32 (25%) HCWs including all 5 HCWs with chronic renal impairment at baseline. Non-surviving HCW had lower PaO2/FiO2 ratios 63.5 (57, 116.2) vs 148 (84, 194.3), p = 0.043, and received more ECMO therapy compared to survivors, 9/32 (28%) vs 4/24 (16.7%) respectively (p = 0.02).Thirteen of the surviving (13/24) HCWs responded to the questionnaire. Two HCWs confirmed functional limitations. Median number of days from hospital discharge until the questionnaires were filled was 580 (95% CI 568, 723.5) days.

**Conclusion:**

Approximately 10% of critically ill patients with MERS were HCWs. Hospital mortality rate was substantial (25%). Patients with chronic renal impairment represented a particularly high-risk group that should receive extra caution during suspected or confirmed MERS cases clinical care assignment and during outbreaks. Long-term repercussions of critical illness due to MERS on HCWs in particular, and patients in general, remain unknown and should be investigated in larger studies.

## Introduction

Middle East Respiratory Syndrome Coronavirus (MERS-CoV) is a beta coronavirus that was first recovered from a patient who died of a fatal pneumonia and multi-organ failure in 2012[1, 2]. Since then, a number of published cohort studies highlighted the variable clinical presentation of MERS, which ranges between minimal or no symptoms and severe, potentially fatal pneumonia that often complicates with multi-organ failure[2–5]. The majority of MERS cases were reported from Saudi Arabia and were linked to healthcare outbreaks[6]. This is most likely related to the overcrowding in emergency departments, lack of diligent application of proper infection control practices, and effective nosocomial transmission[6, 7]. Middle East Respiratory Syndrome coronavirus has been shown to survive the longest in cold and dry environments similar to that of hospitals[8]. Consequently, health care workers (HCWs) were exposed and have been affected with Middle East Respiratory Syndrome (MERS)[9]. Similar to the general population, their clinical presentations ranged from mild or asymptomatic to severe and sometimes fatal MERS[5]. Since the number of critically ill HCWs with MERS in different individual centers is small, adequate data on the impact of the disease in HCWs are lacking. Additionally, the impact of critical illness due to MERS on the long-term functional outcomes of surviving HCWs remains unknown.

We aimed to explore the clinical characteristics and outcomes of critically ill HCWs with MERS. Additionally, we aimed to assess the functional outcomes of surviving HCWs from MERS.

## Methods

### Study design

Data on critically ill HCWs with MERS was extracted from a retrospective multi-center cohort study that was conducted between (0/2012–9/2015) from 14 hospitals in 5 cities across Saudi Arabia. [10]. Physicians and research assistants contacted the surviving HCWs and completed a questionnaire that included a Karnofsky Performance Status (KPS) scale to determine their present post hospital discharge functional outcome (S1) by phone interviews[11]. The functional status would fall in one of three main categories. Performance Status 1 (PS1): able to carry on normal activity and work, no special care needed. Performance Status 2 (PS2): Unable to work, able to live at home, care for most personal needs with a varying degree of assistance needed. Performance Status 3 (PS3): Unable to care for self, requires equivalent of institutional or hospital care [11].

### Institutional Review Board approval

Additional Institutional Review Board approval was obtained from the Institutional Review board of the Ministry of National Guard Health Affairs, Riyadh, Saudi Arabia for interviewing the surviving HCWs and filling the questionnaires. The questionnaire by the HCW was voluntary and included a verbal consent before proceeding with further questions. Participating HCWs were de-identified and study codes were allocated to HCWs by the interviewing research assistants and physicians for data collection and entry.

### Case definition

A HCW is defined as any individual that works in a patient care area in a health care institution.

Critical illness due to MERS was defined as a confirmed MERS-CoV infection with a positive real time MERS-CoV RT-PCR from a respiratory tract sample (nasopharyngeal swab, sputum, deep tracheal aspirate or bronchoalveolar lavage) that led to a critical illness necessitating ICU admission.

The RT-PCR assay used for MERS-CoV diagnosis confirmation targets the upstream of the E protein gene (upE) and the region within open reading frame (ORF)1b as previously described[12].

### Data collection

Data was collected using the international Severe Acute Respiratory and Emerging Infection Consortium (ISARIC) case report. Patients’ demographics including age, gender, underlying comorbid conditions were collected. Clinical features including durations of onset of symptoms to presentation to the emergency room, admission to ICU, and intubation were documented as well. Physiological parameters and laboratory abnormalities were collected. Severity of illness was assessed using the Sequential Organ Failure Assessment (SOFA) scores. Interventions, including medications with antiviral activities and use of steroids, as well as respiratory parameters as well different modes and methods of respiratory support were documented. Outcomes including ICU and hospital mortality were recorded as well.

### Statistical analysis

Descriptive statistics were used for demographics, clinical presentation, parameters as well as interventions and description of outcomes. Fisher’s exact test was used to compare categorical variables and Mann-Whitney *U* test was used to compare continuous variables.

## Results

A total of 32 critically ill HCWs were admitted to ICU with MERS during the study period. The majority of enrolled HCWs were nurses 14/32 (43.75%) followed by physicians 8/32 (25%) (Table 1).

**Table 1:** Baseline characteristics of critically ill healthcare workers (HCWs) with the Middle East Respiratory Syndrome (MERS).

Variable	MERS-CoV N = 32	Survivors N = 24	Non Survivors N = 8	P-value
**Demographics**				
Age (yr), Median (Q1, Q3)	39 (32, 48)	38 (32, 47)	43 (33, 58)	0.35
Male sex, no. (%)	16 (50.0)	13 (54.2)	3 (37.5)	0.69
BMI (Body mass index) (kg/m^2^), Median (Q1, Q3)	30.8 (26.8, 35.2)	30.1 (27.0, 34.0)	38.1 (26.6, 43.7)	0.17
**Comorbidities, n (%)**				
Diabetes with chronic complications	3 (9.4)	1 (4.2)	2 (25.0)	0.15
Chronic cardiac disease	1 (3.1)	1 (4.2)	0 (0.0)	>0.99
Chronic renal disease	5 (15.6)	0 (0.0)	5 (62.5)	0.0003
Chronic pulmonary disease (including asthma)	2 (6.3)	1 (4.2)	1 (12.5)	0.44
Any malignancy including leukemia, lymphoma or solid tumors	2 (6.3)	0 (0.0)	2 (25.0)	0.057
Any comorbidity	11 (34.4)	2 (8.3)	5 (62.5)	0.005
				
Days from onset of symptoms to the emergency room*, median (Q1,Q3)	5.5 (5, 7)	6 (5, 7.0)	5.0 (4.0, 14.0)	0.94
Days from onset of symptoms to ICU admission, median (Q1, Q3)	9 (5, 11)	9.0 (6.0, 11.0)	5 (4.5, 11.0)	0.17
Days from onset of symptoms to intubation, median (Q1, Q3)	9 (6, 12)	10.0 (8.0, 12.0)	6.5 (4.5, 11.0)	0.21
**Respiratory symptoms, n (%)**				
Dyspnea	23 (71.9)	16 (66.7)	7 (87.5)	0.39
Cough	25 (78.1)	20 (83.3)	5 (62.5)	0.33
With sputum	3 (9.4)	3 (12.5)	0 (0.0)	0.55
With bloody sputum	2 (6.3)	2 (8.3)	0 (0.0)	>0.99
Chest pain	6 (18.8)	5 (20.8)	1 (12.5)	>0.99
Sore throat	6 (18.8)	5 (20.8)	1 (12.5)	>0.99
Wheezing	3 (9.4)	3 (12.5)	0 (0.0)	0.55
Rhinorrhea	2 (6.3)	2 (8.3)	0 (0.0)	>0.99
**Gastrointestinal symptoms, n (%)**				
Vomiting / Nausea, n (%)	6 (18.8)	5 (20.8)	1 (12.5)	>0.99
Abdominal pain, n (%)	5 (15.6)	4 (16.7)	1 (12.5)	>0.99
Diarrhea, n (%)	7 (21.9)	6 (25.0)	1 (12.5)	0.64
Gastrointestinal symptoms including nausea, vomiting, abdominal pain or diarrhea	11 (34.4)	8 (33.3)	3 (37.5)	>0.99
**Other symptoms, n (%)**				
Fever (temperature ≥ 38°C), n (%)	30 (93.8)	24 (100.0)	6 (75.0)	0.056
Fatigue, n (%)	9 (28.1)	8 (33.3)	1 (12.5)	0.39
Altered level of consciousness, n (%)	2 (6.3)	1 (4.2)	1 (12.5)	0.44
Myalgia or arthralgia, n (%)	9 (28.1)	7 (29.2)	2 (25.0)	>0.99
Headache, n (%)	**5 (15.6)**	**5 (20.8)**	**0 (0.0)**	0.30

For categorical variables, Fisher’s exact test was used to find the significant difference between the groups.

For continuous variables, Mann-Whitney *U* test was used to calculate *p-*value

### Demographics

Baseline characteristics of the critically ill HCWs with MERS are illustrated in Table 1. Diabetes mellitus was documented in 3/32 (9.4%) HCWs and chronic renal impairment, defined as creatinine clearance of less than 60ml/minute at the time of presentation, was documented in 5/32 (15.6%) HCWs (Table 1). The median body mass index (BMI) was 30.8 (Q1,Q3: 26.8, 35.2), (Table 1). Absence of comorbidities was noted in twenty-one HCWs (65.6%). Two critically ill HCWs were pregnant (Table 1).

### Outcomes

Thirty seven percent (3/8) of the non-surviving HCW were males, median (Q1, Q3) age of the non-surviving HCW was 43 (33, 58). All five HCW who had chronic renal impairment died 5/8 (62.5%). Moreover, of the three of the HCW were known to have diabetes mellitus 3/32 (9.4%), two of which did not survive 2/8 (25%) (Table 1). Both pregnant HCW survived. Non-survivors had significantly lower PaO2/FiO2 ratio compared to survivors 63.5 (57, 116.2) vs 148 (84, 194.3), p = 0.043 (Table 2). Hospital mortality was 8/32 (25%) (Table 3).

**Table 2:** Physiological parameters of critically ill healthcare workers (HCWs) with the Middle East Respiratory Syndrome (MERS).

Variable	MERS-CoV N = 32	Survivors N = 24	Non Survivors N = 8	P-value
**Respiratory parameters on ICU day 1**				
PaO2/FiO2 ratio, median (Q1, Q3)	119.0 (63.5, 176.0)	148.0 (84.0, 194.3)	63.5 (57.0, 116.2)	0.043
PaCO2 (partial pressure of carbon dioxide) (mmHg), median (Q1, Q3)	38.9 (31.9, 49.0)	35.9 (29.0, 42.5)	45.0 (40.5, 53.2)	0.067
PH, median (Q1, Q3)	7.4 (7.3, 7.4)	7.4 (7.4, 7.5)	7.4 (7.2, 7.4)	0.04
Tidal volume (ml), median (Q1, Q3)	404.0 (350.0, 435.0)	392.0 (328.0, 428.0)	427.0 (350.0, 480.0)	0.87
Tidal volume per kg of predicted body weight (ml/kg), median (Q1, Q3)	6.5 (5.4, 8.3)	6.09 (5.7, 8.3)	6.9 (4.2, 9.16)	0.90
PEEP (positive end-expiratory pressure) (cmH_2_0), median (Q1, Q3)	13.0 (10.0, 16.0)	13.0 (10.0, 16.0)	13.5 (10.0, 20.0)	0.77
Plateau pressure (cmH_2_0), median (Q1, Q3)	29.0 (23.0, 30.0)	26.0 (21.5, 31.0)	30.0 (30.0, 30.0)	0.72
Driving Pressure (cmH_2_0), median (Q1, Q3)	13.0 (11.0, 18.0)	12.0 (7.5, 18.0)	18.0 (18.0, 18.0)	0.72
Number of quadrants with infiltrates on chest radiograph, median (Q1, Q3)	2 (2, 3)	2 (2, 3)	3 (2, 4)	0.19
**Extra-pulmonary parameters on ICU day 1**				
Mean arterial pressure (mmHg), median (Q1,Q3)	75.0 (70.0, 85.7)	75.4 (70.0, 89.3)	75 (64.0, 83.0)	0.32
Lactate (mmol/L), median (Q1, Q3)	1.2 (0.9, 1.5)	1.1 (0.9, 1.4)	3.8 (1.3, 6.3)	0.14
Blood Urea Nitrogen (μmol/L), median (Q1, Q3)	2.9 (2.2, 6.4)	2.6 (1.8, 4.1)	9.6 (3.2, 22.3)	0.019
Creatinine (μmol/L), median (Q1, Q3)	69.0 (60.0, 91.0)	63.5 (55.5, 73.5)	132.6 (81.0, 265.2)	0.001
Hemoglobin g/dL, median (Q1, Q3)	12.9 (11.2, 13.9)	13.3 (12.1, 14.2)	10.8 (8.9, 12.7)	0.017
Platelets (x10^9^/L), median (Q1, Q3)	179.0 (123.0, 198.0)	180.0 (142.5, 209.0)	123.0 (64.0, 181.0)	0.18
Bilirubin (μmol/L), median (Q1, Q3)	10.0 (5.9, 13.9)	9.4 (5.6, 12.0)	12.0 (7.0, 50.8)	0.23
ALT (Alanine amino transferase) (U/L), median (Q1,Q3)	49.6 (30.0, 104.0)	49.3 (32.0, 104.0)	67.7 (20, 111.0)	0.81
GCS (Glasgow Coma Scale), median (Q1, Q3)	15.0 (3.0, 15.0)	15.0 (4.0, 15.0)	4.5 (3.0, 9.0)	0.035
SOFA score (Sequential Organ Failure Assessment), median (Q1, Q3)	4.0 (3.0, 8.0)	3.5 (2.0, 6.5)	8.0 (4.0, 8.0)	0.086

Mann-Whitney *U* test was used to calculate *p-*value

**Table 3:** Main interventions and outcomes in critically ill healthcare workers (HCWs) with the Middle East Respiratory Syndrome (MERS).

Variable	MERS-CoV N = 3232	Survivors N = 24	Non Survivors N = 8	P-value
**Management**				
Non-invasive positive pressure ventilation, n (%)	13 (40.6)	9 (37.5)	4 (50.0)	0.68
Invasive ventilation, n (%)	26 (81.3)	18 (75.0)	8 (100.0)	0.29
Nitric oxide, n (%)	5 (15.6)	4 (16.7)	1 (12.5)	>0.99
Prone positioning, n (%)	6 (18.8)	5 (20.8)	1 (12.5)	>0.99
High-frequency oscillation ventilation, n (%)	3 (9.4)	1 (4.2)	2 (25.0)	0.15
ECMO (extracorporeal membrane oxygenation), n (%)	9 (28.1)	4 (16.7)	5 (62.5)	0.02
Vasopressors, n (%)	19 (59.4)	12 (50.0)	7 (87.5)	0.1006
Renal replacement therapy, n (%)	10 (31.3)	6 (25.0)	4 (50.0)	0.22
Blood transfusion, n (%)	9 (28.1)	5 (20.8)	4 (50.0)	0.18
Tracheostomy, n (%)	1 (3.1)	0 (0.0)	1 (12.5)	0.25
**Medications, n (%)**				
Antivirals, n (%)	32 (100)	24 (100.0)	8 (100.0)	-
Oseltamivir, n (%)	27 (84.4)	22 (91.7)	5 (62.5)	0.085
Both Ribavirin and interferon, n (%)	13 (40.6)	9 (37.5)	4 (50.0)	0.76
Interferon only, n (%)	0 (0.0)	0 (0.0)	0 (0.0)	
Ribavirin only, n (%)	1 (3.1)	1 (4.2)	0 (0.0)	
**Other medications**				
Corticosteroids, n (%)	13 (40.6)	7 (29.2)	6 (75.0)	0.038
Intravenous immunoglobulin, n (%)	3 (9.4)	3 (12.5)	0 (0.0)	0.55
**Outcomes, n (%)**				
ICU mortality, n (%)	6 (18.8)	0 (0.0)	6 (75.0)	0.0001
ICU length of stay (days), median (Q1, Q3)	12 (5, 21)	13.5 (6.0, 24.5)	8.0 (5.0, 12.0)	0.20
Hospital mortality, n (%)	8 (25.0)	0 (0.0)	8 (100.0)	<0.001
Hospital length of stay (days), median (Q1, Q3)	22.5 (14.0, 56.0)	25.5 (17.5, 56.0)	15.5 (8.0, 65.5)	0.22

For continuous variables, Mann-Whitney *U* test was used to calculate *p-*value. For categorical variables, Fisher’s Exact test was used to calculate *p* values.

### Clinical features

Affected HCWs presented to emergency department (ED) within the first week of their symptoms manifesting, at a median (Q1, Q3) of 5.5 days (5, 7). They required intubation within a median of 9 (6, 12) days of the onset of their symptoms. Cough was notably absent in 7/32 (22%) HCWs. Eleven (34%) HCWs presented with gastrointestinal symptoms. Fever was documented in 30/32 (93.8%) HCWs on presentation (Table 1).

Median serum creatinine was significantly higher in non-survivors: 132.6 μmol/L (81, 265.2) compared to that of survivors 63.5 (55.5, 73.5) (p = 0.001) (Table 2). Other physiologic parameters are shown in Table 2.

### Interventions

Eighty-one percent (26/32) HCWs required intubation and mechanical ventilation. Nitric oxide, high-frequency oscillation ventilation and ECMO were used as rescue oxygenation therapy in 5/32 (15.6%), 3/32 (9%) and 9/32 (28%) respectively. Sixty-three percent (5/8) of non-survivors received ECMO compared to 16.7% of survivors (4/24) (p = 0.02) (Table 3).

Hemodynamic instability that necessitated the use of vasopressor support occurred in 19/32 (59.4%) HCWs, and renal replacement therapy was instituted in 10/32 (31.3%) HCWs (Table 3).

Antiviral therapy that includes interferon and/or ribavirin were used in a total of 43.8% (14/32) HCWs, whereas oseltamivir was used empirically as part of management of severe acute respiratory infection in 84% (27/32) HCWs pending influenza PCR results. No significant differences were noted between survivors and non-survivors in the use of potentially MERS-CoV active antiviral agents, namely interferon and ribavirin (Table 3).

### Performance scores

The 24 surviving HCWs were contacted by phone to complete the performance scores questionnaire. Thirteen (13/24) HCWs responded. Median number of days from hospital discharge until the questionnaires were filled was 580 (IQR 568, 723.5) days. Three HCWs had left the country and were unreachable. Five did not answer when were contacted for the questionnaire, and 3 HCW did not give consent to participate. Of the respondents, 11/13 HCW scored PS1 (Able to carry on normal activity and to work, no special care needed) on Karnofsky Performance Scale. Two HCW scored 2 on the Karnofsky Performance Scale (Unable to work, able to live at home and care for most personal needs with varying amounts of assistance needed) (Table 4). The first HCW was a 34-year old female nurse, with no comorbid conditions, who worked in the ICU and was 32 weeks pregnant at the time of diagnosis with MERS. The recovering HCW stated that she has developed physical limitations in terms of inability to carry her toddler son, walking for distances longer than 30 minutes and climbing more than 1 flight of stairs. Upon asking whether MERS affected her career she responded that she requested to be transferred to an administrative job that is not physically demanding and requires less working hours. She had returned to work 270 days after hospital discharge. The second HCW was a 29-year old physician who had no comorbid conditions. He returned to work 120 days after hospital discharge. Both HCWs required invasive mechanical ventilation during their hospital stay. Of note, both HCWs abstained from work the longest after hospital discharge, 270 and 120 days respectively. The remaining 11 HCW, who responded to the questionnaire, resumed working after a median of 30 (IQR 22, 45) days after hospital discharge.

**Table 4:** Questionnaire data for healthcare workers (HCWs) who survived critical illness due to the Middle East Respiratory Syndrome (MERS).

Variable	N 13 (%)
Male sex, no. (%)	8 (62)
Age, Median (Q1, Q3)	35 (30.5, 47.5)
**Nationality**	
Saudi, no. (%)	4 (31)
Others, no. (%)	9 (69)
**Occupation**	
Physician, no. (%)	4 (31)
Nurse, no. (%)	6 (46)
Respiratory therapist, no. (%)	1 (8)
Other, no. (%)	2 (15)
**Location of work**	
Emergency room, no. (%)	4 (31)
Ward of confirmed cases, no. (%)	3 (23)
Other, no. (%)	6 (46)
Time to return to work (days), median (Q1, Q3)	30 (30, 75)
**Karnofsky Performance Scale (PS)**	
PS1 (Able to work-normal activity), no. (%)	11 (85)
PS2 (Unable to work-cares for most personal needs), no. (%)	2 (15)
PS3 (Unable to care for self-requires equivalent of institutional care), no. (%)	0 (0)
**Perceptions of affected HCWs**	
Infection avoidable by complying to infection control precautions (Yes), no. (%)	11 (85)
Change in infection control practices after recovery (Yes), no. (%)	11 (85)

## Discussion

Middle East Respiratory Syndrome Coronavirus continues to affect patients with severe and potentially fatal pneumonia. Effective nosocomial transmission and consequent hospital outbreaks posed a major challenge on HCWs. A number of single center MERS cohort studies included affected HCWs[5, 13]. The numbers of included HCWs, however, in each of those studies are very few, and some of whom had minimal or no symptoms. This, to our knowledge, is the only multi-center cohort study of critically ill HCWs with MERS.

Our study demonstrates that hospital mortality among critically ill HCWs with MERS was substantial [25% (8/32)], but lower that what has been reported in general ICU MERS patients. A recent published cohort of 330 confirmed critically ill MERS cases (inclusive of the current HCWs cases) demonstrated hospital mortality of 67% (223/330) [10]. This is probably related to the relatively younger age of the HCWs cohort compared to that of the general population MERS SARI cohort [median age (Q1, Q3) of 39 (32, 48) compared to 58 (44, 69)]. Additionally, HCWs were less likely to have comorbid conditions (34.4% compared to 80.3%). Advanced age has been consistently recognized as a risk factor for mortality in several MERS cohort studies[3, 5, 14]. Additionally, presence of comorbid conditions, particularly diabetes mellitus as well as renal failure has been shown to be significantly associated with mortality in confirmed MERS cases[5, 14]. Interestingly, none of the five HCWs who were known to have renal impairment at baseline survived. This is consistent with previous cohort studies where patients with renal failure, and patients who required hemodialysis have had a significantly increased mortality rate reaching 100%[5]. Immune dysregulation has been studied and documented in patients with chronic renal impairment and those with end stage renal diseases who required hemodialysis[15]. Reduced T-cell quantity as well as helper T-cell responses to mitogen stimulation has been documented and in some studies linked to durations of hemodialysis and renal impairment[15, 16]. This immune dysfunction may therefore explain the witnessed increased mortality rates in MERS patients with chronic renal impairment.

The noted increased use of ECMO in non-surviving HCW is probably reflective of the severity of respiratory failure where ECMO is used as a rescue therapy.

Our cohort included two pregnant women who developed critical illness due to MERS and survived.

In a previously published cohort of five pregnant women with MERS, all were admitted to ICU and two patients died, one of who was known to have bronchial asthma, lung fibrosis and spontaneous pneumothoraces. Only one of the pregnancies resulted in intrauterine fetal demise. The remaining 3 pregnant HCWs survived.[17] There are no data however, on their long-term functional outcomes. Although the two pregnant women in our cohort have survived MERS, one HCW did indicate limitations in her physical ability after recovery from MERS, whereas the second was an expat who has left the country and was unreachable. It is therefore, not possible to conclude whether pregnancy correlates with MERS severity or poorer functional outcomes. In a case control study, pregnant women with confirmed SARS had more severe pneumonia and worse outcomes[18]. On the other hand, data were conflicting on whether outcomes of H1N1 pneumonia were worse in pregnant compared to non-pregnant women[19, 20].

Interestingly, Eleven (11/13) of the responding HCWs thought that MERS-CoV infection could have been avoided had they complied with infection control precautions and wearing personal protective equipment (Table 3). A similar number (11/13) reported a change in their infection control practices in a shift towards increased compliance. Examples of those changes in practices consistently cited compliance to personal protective equipment when advised particularly when dealing with suspected or confirmed MERS cases.

We selected Karnofsky Performance Scale to assess functional outcomes of surviving HCW from MERS. Having been originally developed by Karnofsky to assess the functional ability and prognosis in patients with malignancies, the elements included in the score assessed for physical ability to determine whether the tested individual is able to carry out normal activities without any reference to a specific oncologic diagnosis, size of a tumor or type of chemotherapy[11]. It therefore, has been used assess functional status of patients of other non-oncologic conditions. Compared to other quality of life models, KFS does not dwell into social, economical and spiritual factors, which are out of the scope and the purpose of this questionnaire which focuses on perceiving whether relatively young and healthy MERS SARI survivors could have long term physical effects of their illness[21]. Additionally, other tests such as physical performance test are designed to assess physical ability of elderly patients and scores are based on specific physical tests that the patient is required to perform which would not have been feasible in our setting [22]. While recall bias poses a possible study limitation for questions related to the exposure, it does not affect the answers to the functional assessment questions, which inquire about the status at the time of the questionnaire. In fact, persistence of fatigue or exertional shortness of breath over a year or longer after discharge highlights the seriousness of such symptoms. Indeed, the self-reported post recovery functional limitation in two previously healthy and young HCWs are very worrisome and warrants investigation. This is particularly true since HCWs were approached after a median of 580 days from hospital discharge. The fact that those HCW continued to experience persistent change in exercise capacity highlights an overlooked complication of MERS, and that death is not the only negative outcome in affected patients. A previous report highlighted similar concerns in SARS survivors[23].

Despite the fact that the questionnaire was short and took a few minutes to complete by HCWs who participated, three have declined and five have not answered calls by study coordinators. Declining to answer a short questionnaire that may serve as a reminder of an unpleasant and a possibly traumatic experience may indicate unresolved stress or post-traumatic stress disorder, particularly that the reason behind this experience is directly related to their profession in health care. A previous report on HCWs with MERS highlighted the stress and negative feelings associated with the contracting the infection, however it was in a group of HCWs in a hospital during a nosocomial outbreak where no HCW died [24]. Previous reports of increased rates of depression and post-traumatic stress disorder in HCWs, who were considered at high risk for contracting SARS illnesses, even in the absence of an actual infection, during the year that followed the SARS outbreak were published [25]. Our interpretation, however, remains speculative and a formal psychological assessment is needed.

Moreover, outbreaks such as that of MERS can impose a huge strain on hospital resources in several ways. For instance, HCWs who are suspected to have MERS should abstain from work until they are tested negative. In addition, contact screening of HCWs for MERS following contact with a confirmed or a suspected case can lead to over testing, which can be expensive and labor intensive. A previous report of a confirmed MERS in a HCW has led to performing over 150 tests over 3 days[9].

Lack of response from 11 HCW is a limitation in our study particularly that five of those HCW were expats who have left the country without means of communicating with them. Whether those HCW performed worse on Karnofsky Performance Scale or had residual cognitive impairment is unknown and cannot be determined. The retrospective nature of our study is another limitation.

## Conclusions

In conclusion, this is the largest cohort of critically ill HCWs with MERS to date. The lower mortality rates, younger age and fewer comorbidities in comparison to the general population are highlighted in this study. Nevertheless, MERS related mortality in HCWs remained substantial, particularly in HCWs with chronic renal impairment. This sheds a new light on a specifically high-risk group that should be offered special attention especially during clinical care assignment to suspected or confirmed MERS cases. Additionally, the often overlooked residual physical and emotional negative outcomes need to be systematically assessed and addressed in a timely manner by health care institutions. Failure to do so may negatively impact the working environment, which would affect quality of care provided to patients particularly during and after periods of outbreaks.

In the absence of effective therapies or vaccines, it is crucial to train HCWs to recognize possible MERS cases and to strictly implement and comply with infection control precautions particularly when dealing with suspected or confirmed MERS cases.

## Supporting information

S1 File Supplement table (S1). (DOCX)

S2 File HCW data set (S2). (RAR)

## Abbreviations

MERS-CoV: Middle East Respiratory Syndrome Coronavirus
HCWs: Health care workers
ISARIC: international Severe Acute Respiratory and Emerging Infection Consortium
SARI: Severe Acute Respiratory Infection
ICU: intensive care unit
KPS: Karnofsky Performance Status
NP swab: nasopharyngeal swab
RT-PCR: reverse transcription polymerase chain reaction
SOFA: Organ Failure Assessment
ED: Emergency department
ECMO: Extracorporeal membrane oxygenation
SARS: severe acute respiratory tract syndrome
