# Supplementary material for: Critically ill healthcare workers with the middle east respiratory syndrome (MERS): A multicenter study
Source: PLoS One. 2018 Nov 15;13(11):e0206831. doi: 10.1371/journal.pone.0206831 (PMC6237307; doi:10.1371/journal.pone.0206831)
Supplement: S1 File — (DOCX) [file pone.0206831.s001.docx]

**Supplementary to**

**Critically Ill Healthcare Workers with the Middle East Respiratory Syndrome (MERS): A Multicenter Study**

**Contents:**

**Collaborators**

**Supplement to Methods**

**S1 Table:** Health care workers questionnaire

**Collaborators**

| Center | Names |
| --- | --- |
| **Saudi Arabia-The Saudi Critical Care Trials group** |  |
| King Saud bin Abdulaziz University for Health Sciences and King Abdullah International Medical Research Center, Riyadh | Yaseen Arabi  Abdulaziz Aldawood  Hanan Balkhy  Mohamed A. Hussein  Mashael Al Ahmadi  Musharaf Sadat  Eman Al Qasim  Hasan Aldorzi  Jesna Jose |
| King Fahad Medical City, Riyadh | Abdullah Al Motairi |
| Prince Sultan Cardiac Center, Riyadh | Ghaleb A. Almekhlafi  Yasser Mandourah  Sahar Hassan  Abid Alwan  Raylin Cabal  Rima E Mahamed  Khaloud M. Harbi  Abdulelah Ala Haidary |
| King Saud Medical City, Riyadh | Abelrahman Al-Harthy  Ahmed Fouad Mady   Omar Elsayed Ramadan  Muhammed Asim Rana  Basim Raafat Huwait  Mohamed Ali Al-Odat  Waleed Tharwat Al-Atreeby |
| King Faisal Specialist Hospital and Research Centre, Riyadh | Othman Solaiman |
| King Khalid University hospital, Riyadh | Ahmed Abdul Mommin  Muhammed Fares  Mazen Barry |
| Security Forces Hospital, Riyadh | Awad Al Omari |
| King Abdulaziz Medical City, Jeddah | Fahad Al-Hameed  Jalal Al Refai |
| King Fahd Armed Forced Hospital, Jeddah | Sarah Shalhoub |
| King Faisel Hospital Research Center, Jeddah | Basem M Alraddadi  Rashed E Alrehaili  Sarah Batawi |
| King Fahad Hospital Group, Jeddah | Rajaa Al-Raddadi  Ahmed Rajab  Omimah Shabouni  Abeer Mustafa Housa  Amal AbdulallaTurkistani  Abdullatif Ayesh Almarashi  Amaal Ali Sarraj  Salwa Awad Own  Sara Mohammed AlJeaid  Wijdan Abdulalkareem Baeshen |
| AlNoor Specialist Hospital, Makkah | Kasim Al Khatib  Hamdy Badr  Majduldeen Azzo |
| King Abdulaziz Medical city-Alahsa | Abdulsalam Alaithan |
|  |  |
| King Fahad Hospital, Madinah | Ayman Kharaba  Noah Noor |
| **United Kingdom** |  |
| Infectious Diseases Data Observatory, Oxford University | Laura Merson |
| **Canada** |  |
| Sunnybrook Health Sciences Centre, Canada | Robert Fowler  Ruxandra Pinto |
| **United States of America** |  |
| University of Virginia School of Medicine, USA | Frederick Hayden |
|  |  |

**Supplement to Methods**

**S1 Table:** Health care workers questionnaire

| Personal Data: | |  | |  | |
| --- | --- | --- | --- | --- | --- |
| Study Number ___________________ | | Name: | | | |
| Gender: | | | | Age: | |
| Nationality: | |  | | | |
|  | |  | |  | |
| Questions: | |  | |  | |
|  | |  | |  | |
| 1-What is the nature of your work? | | | | | |
|  Doctor | | | | | |
|  Nurse | | | | | |
|  Laboratory personnel | | | | | |
|  other, Specify: | | | | | |
|  | |  | |  | |
| 1-What was the type of exposure? | | | |  | |
|  | Close contact (closer than 1.5 meters for ≥ 10 minutes) | | |  | |
|  | Non- close contact | | |  | |
|  |  Others if yes specify: | | | | |
|  |  | | | |  |
| 2-How long did you take care of patient? | | | | |  |
|  |  More than 24 hours | |  | | |
|  |  24 hours or less | | No. of hours: | | |
|  |  | | |  | |
| 3-Where was your location of work | | | | | |
|  |  ER | | | |  |
|  |  floor of admitted confirmed cases | | | |  |
|  |  floor of admitted suspected cases | | | |  |
|  |  other, if yes specify: | | | | |
| 4- Date of return to work | | | | | |
| Date of discharge from the hospital | | | | | |
| 5- Post discharge functional status (Karnofsky Performance Status Scale) | | | | | |
| Able to carry on normal activity and to work; no special care needed (PS I)   No  Yes | | | | | |
| Unable to work; able to live at home and care for most personal needs;  varying amount of assistance needed (PS II)  No  Yes | | | | | |
| Unable to care for self; requires equivalent of hospital care;  (PS III)  No  Yes | | | | | |
|  | | | | | |
| 6- Do you think that your infection could have been avoided by applying infection control measures? | | | | | |
|  No  Yes | | | | | |
| Comments: | | | | | |
| 7- Has it affected your infection control practices?  No  Yes  If yes, explain? | | | | | |
